# T-S2Inet: Transformer-based sequence-to-image network for accurate nanopore sequence recognition

**DOI:** 10.1093/bioinformatics/btae083

**Published:** 2024-02-15

**Authors:** Xiaoyu Guan, Wei Shao, Daoqiang Zhang

**Affiliations:** College of Computer Science and Technology, Nanjing University of Aeronautics and Astronautics, MIIT Key Laboratory of Pattern Analysis and Machine Intelligence, Nanjing 211106, China; Key Laboratory of Brain-Machine Intelligence Technology, Ministry of Education, Nanjing University of Aeronautics and Astronautics, Nanjing 211106, China; College of Computer Science and Technology, Nanjing University of Aeronautics and Astronautics, MIIT Key Laboratory of Pattern Analysis and Machine Intelligence, Nanjing 211106, China; Key Laboratory of Brain-Machine Intelligence Technology, Ministry of Education, Nanjing University of Aeronautics and Astronautics, Nanjing 211106, China; College of Computer Science and Technology, Nanjing University of Aeronautics and Astronautics, MIIT Key Laboratory of Pattern Analysis and Machine Intelligence, Nanjing 211106, China; Key Laboratory of Brain-Machine Intelligence Technology, Ministry of Education, Nanjing University of Aeronautics and Astronautics, Nanjing 211106, China

## Abstract

**Motivation:**

Nanopore sequencing is a new macromolecular recognition and perception technology that enables high-throughput sequencing of DNA, RNA, even protein molecules. The sequences generated by nanopore sequencing span a large time frame, and the labor and time costs incurred by traditional analysis methods are substantial. Recently, research on nanopore data analysis using machine learning algorithms has gained unceasing momentum, but there is often a significant gap between traditional and deep learning methods in terms of classification results. To analyze nanopore data using deep learning technologies, measures such as sequence completion and sequence transformation can be employed. However, these technologies do not preserve the local features of the sequences. To address this issue, we propose a sequence-to-image (S2I) module that transforms sequences of unequal length into images. Additionally, we propose the Transformer-based T-S2Inet model to capture the important information and improve the classification accuracy.

**Results:**

Quantitative and qualitative analysis shows that the experimental results have an improvement of around 2% in accuracy compared to previous methods. The proposed method is adaptable to other nanopore platforms, such as the Oxford nanopore. It is worth noting that the proposed method not only aims to achieve the most advanced performance, but also provides a general idea for the analysis of nanopore sequences of unequal length.

**Availability and implementation:**

The main program is available at https://github.com/guanxiaoyu11/S2Inet.

## 1 Introduction

Nanopore sequencing technology is a newly developed approach for macromolecular sensing and recognition, which facilitates the sequencing of DNA (or RNA) and other molecules, enables the detection of methylation, and can even be utilized for data storage (Kasianowicz *et al.* 1996, Ying *et al.* 2014). It is considered the cutting-edge third-generation sequencing platform because of its extended read times for macromolecules and precise resolution of single bases. Typically, nanopore sequencing devices consist of liquid-filled reservoirs interconnected by a single nanopore (Henley *et al.* 2016). The upper and lower parts of the reservoir involve a potential difference. Upon entry of the molecules into the sequencing apparatus, the potential difference propels them toward the lower potential and into the nanopores. At this point, a blocking current generates molecular properties that reflect the physical, chemical, and structural characteristics of the analyzed molecule (Aksimentiev *et al.* 2004, Majd *et al.* 2010, Steinbock *et al.* 2014).

In general, nanopore sensors can be classified into two categories based on their construction materials: solid-state nanopores and biological nanopores. Solid nanopores are constructed of solid materials that can be mass-produced through semiconductor fabrication (Feng *et al.* 2015). They are widely used for DNA sequencing and protein detection due to their cost-effectiveness and scalability (Traversi *et al.* 2013). On the other hand, biological nanopores are composed of transmembrane protein channels that can recognize and sequence individual molecules (Smith *et al.* 2015, Zhang *et al.* 2015, Zhang *et al.* 2017). One of the most commonly employed biosensors in the academic sphere is the *Mycobacterium smegmatis* porin A (MspA) transmembrane protein channel, which presents a rigid *β*-barrel structure. This nanopore has been utilized to analyze RNA tertiary structures at the single-molecule level (Wang *et al.* 2021). Furthermore, it can directly distinguish many low molecular weight RNA structures, including miRNA, overhanged siRNA, blunt siRNA, tRNA, and 5S rRNA. In the industrial sector, Oxford Nanopore Technology (ONT) has developed a line of portable, affordable, and user-friendly nanopore sensors (Laver *et al.* 2015, Hoenen *et al.* 2016, Loose *et al.* 2016, Castro-Wallace *et al.* 2017, Johnson *et al.* 2017). Among these, the MinION is a USB-powered device that is 4 inches in length and contains 512 sensor arrays, each of which is linked to four biological nanopores integrated into a resistive artificial membrane.

Indeed, nanopore sequencing technology plays a significant role in whole human genome sequencing. Unfortunately, this technology generated sequence is subject to signal bias, making manual processing of the sequence data challenging. However, with advances in computing power and machine learning, such as Convolutional Neural Networks (CNN), Support Vector Machine (SVM), or Random Forest (RF), it is now possible to analyze long nanopore read sequences. Machine learning algorithms used in the nanopore domain can be classified into traditional and deep learning methods. For instance, RF and SVM were used to process ionic current signals obtained from solid-state nanopore sequencing of polypeptide chains, displaying the sensitivity of sub-nanopore-derived signals in recognizing protein sequences (Kolmogorov *et al.* 2017). Jia *et al.* (2019) trained an SVM classifier to detect DNA methylation events from raw data, whereas Schreiber et al. proposed a Hidden Markov Model that could segment and integrate nanopore data (Schreiber and Karplus 2015). Furthermore, Liu *et al.* (2019) developed an SVM classifier for accurately detecting N6-methyl adenosine (m6A) RNA changes in nanopores. On the other hand, Farshad and Rasaiah (2020) designed a two-layer neural network with Levenberg Marquardt (LM) transfer function to analyze Silicon Nitride (Si_3_N_4_) nanopore data, while Ni *et al.* (2019) leverage deep learning techniques to detect DNA methylation status from nanopore sequencing. Lastly, Liu *et al.* (2019) employed deep recurrent neural networks to detect DNA base modifications in Oxford nanopore sequencing data.

These machine learning algorithms have enabled unprecedented breakthroughs in the application of nanopore sequencing to various biological tasks. In comparison to traditional machine learning methods, deep learning methods have achieved significant success in performance. Deep learning methods have been successfully implemented in various application areas, including computer vision (CV) (Zhou *et al.* 2021, Zou *et al.* 2022, Zhao *et al.* 2023), natural language processing (NLP) (Sun *et al.* 2022), and various data analysis tasks (Qiao *et al.* 2023, Wang *et al.* 2023). However, while deep learning is processing sequence data, the data input to the network must be of the same length. When nanopore sequencing involves a single molecule, such as sequencing RNA molecules (Wang *et al.* 2021), the sequencing time of each molecule is not the same, generating nanopore data of unequal length. For the eight types of barcode classification tasks generated by ONT, sequences can be transformed into sequences of the same length using interpolation technology due to the relatively small length differences between them. Nevertheless, RNA molecule classification data may witness a length difference of up to 100 000, making it hard to process using interpolation technology. To enable deep learning technology to be applied to this nanopore sequencing data, a sequence transformation module that preserves pivotal sequence features needs to be devised with caution. A method similar to histogram mapping, used to propose the S2Snet network that achieved 95.1% accuracy for RNA classification datasets, was previously attempted (Guan *et al.* 2022). However, S2Snet is a one-dimensional deep learning network for sequences that cannot efficiently extract vital regional features. Therefore, we test whether converting a one-dimensional sequence into a two-dimensional image can aid in using the state-of-the-art Transformer mechanism to extract critical features in the sample.

The sequential data generated by nanopores, which is one-dimensional and of unequal lengths, needs to be transformed for deep learning network input. In order to extract characteristics more effectively, the one-dimensional sequence must be transformed into two-dimensional image data, which can be accomplished in multiple ways, including the use of Gramian Angular Fields (GAFs) (Zhang *et al.* 2019), Markov Transition Field (MTF) (Zhang *et al.* 2018), Recurrence Plot (RP) (Marwan *et al.* 2002). GAFs remodel the one-dimensional sequence data from a rectangular coordinate system to a polar coordinate system and then correlate different sequence points by examining their angle sum or difference. This method is split into Gramian Angular Summary Field (GASF) (corresponding to the angle sum) and Gramian Angular Difference Field (GADF) (corresponding to the angle difference). MTF is built on first-order Markov chain analysis to incorporate temporal relations into the Markov transfer matrix. Nonetheless, MTF is inapplicable to nanopore sequence data since it exclusively analyzes sequence morphology and is not sensitive to temporal relations. RP is an integral method for scrutinizing the periodicity, chaos, and nonstationarity of time series. It reveals the internal structure of the time series and furnishes prior knowledge about similarity, informational content, and predictability. However, the recursive graph is better suited for short time series data, while nanopore sequences can extend up to several million timepoints. Therefore, GAFs are a better option for converting nanopore sequences into image data.

In regards to image data, the Transformer framework (Vaswani *et al.* 2017) has notable advantages over CNN and has shown promising results on NLP tasks in recent years. The initial adoption of Transformer in CV was primarily for extracting information from sequences, such as certain video tasks. However, the ViT approach (Zhou *et al.* 2021) has expanded the scope of Transformer due to its superior global properties. One limitation of CNNs is their relatively restricted receptive field. To expand the receptive field of the network, a structure of convolution-pooling-stacked layers is necessary. Nevertheless, the receptive field decreases outward in a Gaussian manner with a particular center as the reference point. Consequently, CNNs tend to concentrate on only one or two significant parts of the image (Zhang *et al.* 2016). To address this issue, the S2Snet approach (Guan *et al.* 2022) employs an attention mechanism. On the other hand, Transformer can leverage global effective information, and the multi-head mechanism guarantees that the network can focus on various critical areas, each with independent attention. Accordingly, in this article, we utilize Transformer to process image data after converting the nanopore sequence.

Therefore, we propose a sequence-to-image (S2I) module based on GAFs for transforming one-dimensional sequences into two-dimensional images. Additionally, we have designed a Transformer-based network structure, T-S2Inet, to implement this transformation module. The performance evaluation was conducted using RNA molecular classification dataset and eight kinds of barcode classification tasks generated by the ONT equipment. The key contributions of this article are:

- First, we propose a novel S2I module that converts nanopore data into images, thereby enabling the use of existing deep learning techniques.
- Second, we present T-S2Inet, which is based on the S2I module. T-S2Inet uses the Transformer framework to capture the vital image feature information.
- To evaluate the effectiveness of our proposed method, experiments with nanopore RNA datasets and ONT barcode datasets demonstrate that it has the best performance and efficiency.

## 2 Preliminaries

Before presenting our T-S2Inet deep neural network model, which is based on a S2I transformation module, this section will briefly introduce two concepts that readers should be familiar with: (1) addressing the issue of unequal lengths of nanopore sequences and potential solutions, and (2) introducing the transformer mechanism.

### 2.1 Nanopore problem statement

Before delving into the analysis of nanopore data, it is imperative to understand the pertinent concepts associated with it. Evidently, nanopore data is a continuous time series, thereby facilitating the application of various time series processing techniques in its analysis. In the context of RNA type prediction, the data are derived from sequencing six RNA molecules using nanopore devices.

**Figure 1.**
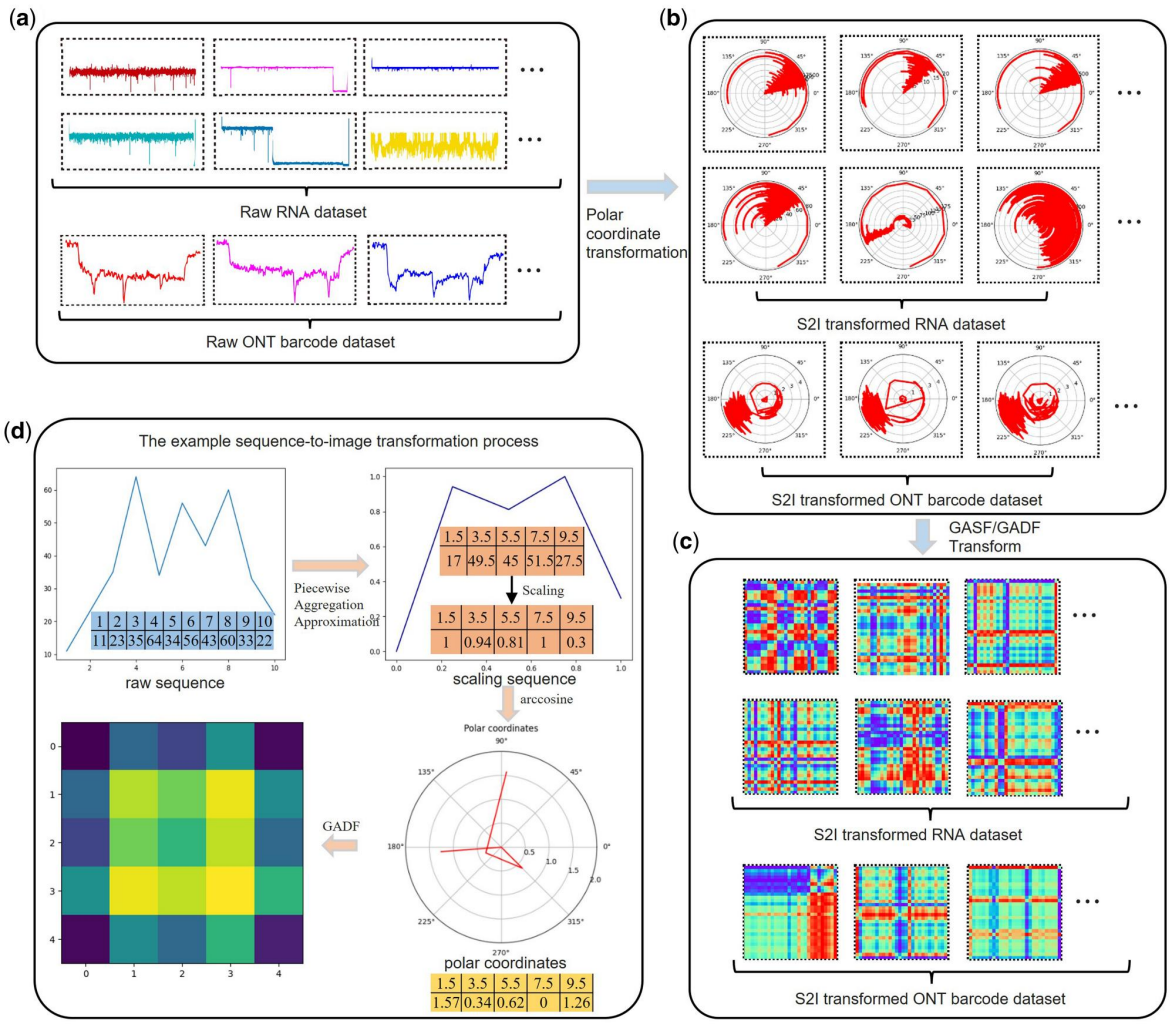
The example samples from the RNA dataset and ONT barcode dataset. (a) The example samples from the Raw RNA nanopore dataset and Raw ONT barcode nanopore dataset, different colors represent different categories. (b) The example samples of the after polar coordinate transformation process. (c) The example samples from the Transformed RNA dataset and Transformed ONT barcode dataset by the S2I module. (d) The example sequence-to-image transformation process.

In general, supposing that the symbols *S* represent the original time series and *T* represent the RNA types. The input long sequence *S* is truncated to *n* sub-sequence $s=[s_{1},{\;s}_{2},\;\cdots ,{\;s}_{n}$], and the input RNA type *T* is truncated to *n* sub-targets $t=[t_{1},\;t_{2},\;\cdots ,\;t_{n}$]. It considers that the previous paper used the RF algorithm as the classification model to distinguish the RNA types (Wang *et al.* 2021, Guan *et al.* 2022). Therefore, the input of RF algorithm is the feature vector (***v***_*i*_) of the sub-sequence *s_i_* extracted by feature extract methods, which contains the length, mean, standard deviation and other statistical information. Traditionally, the RNA types prediction problem can be defined as a classification task:

(1) $$f:(v_{i},t_{i})\to y$$

where *y* is the predicted RNA type, and *f* is the mathematically rigorous function mapping (i.e. the RF algorithm for the problem of RNA types prediction). For the RNA type prediction task, the data come from the MspA nanopore, and the collected data include the detection signals of overhung siRNA, blunt siRNA, tRNA, and 5S rRNA, as well as noise signals. The length of the detection signal corresponding to each RNA molecule is not strictly equal (as shown in Fig. 1a), which leads to its inability to directly input into the deep learning model.

In contrast, the data type of the ONT barcode classification problem is the same (Bell and Keyser 2016, Misiunas *et al.* 2018). Different codes are defined according to the morphology of different waveforms (as shown in Fig. 1a). Different sequence segments also have mismatched sequence lengths. However, the classification standard is different because it is not governed by the number of the current signal peaks in the ONT barcode data. For example, the first peak marks the beginning, three bits of uniquely identification molecules are designed, and the last peak indicates the end. The whole dataset has eight types of barcodes from “000” to “111”. Misiunas *et al.* (2018) applied the CNN model to solve the specific problem, which can automatically extract features to distinguish the barcode types. Therefore, for an *L*-layer CNN model, the ONT barcode classification problem can be defined as:

(2) $$x_{l}=f^{\left(l\right)}(x_{l-1};\theta_{l}),l=1,\ldots ,L$$

where *θ_l_* is the parameters of the *l*th layer and $f^{\left(l\right)}(x_{l-1};\theta_{l})$is the mathematically rigorous mapping function of the *l*th layer. The *x_l_* is the output of *l*th layer, and the *x_l-1_* is the input of (*l-*1)th layer.

### 2.2 Transformer mechanism

The Transformer model finds application in the field of NLP for sequence translation. Transformer model adopts encoder decoder architecture. The Encoder-Decoder of Transformer includes *Add* module, *Norm* module and Multi-Head Attention module. *Add* module indicates residual connection, which is used to prevent network degradation, and *Norm* indicates layer normalization, which is used to normalize the activation value of each layer. The most important module is Multi-Head Attention, which is composed of multiple Self-Attention, so we focus on Self-Attention this Section. For the structure of Self-Attention, matrices **Q** (query), **K** (key value) and **V** (value) need to be used in calculation. The input of Self-Attention is represented by matrix *X*, and **Q**, **K** and **V** can be calculated by using linear variable matrix **WQ**, **WK** and **WV**. After obtaining the matrices **Q**, **K** and **V**, the output of Self-Attention can be calculated. The calculation formula is as follows:

(3) $$\mathrm{Attention}\left(Q,\;K,\;V\right)=\mathrm{softmax}(\frac{QK^{T}}{\sqrt{d_{k}}})V$$

where $.^{T}$ represents transposition, $d_{k}$ is the number of columns of **Q**, **K** matrix, that is, the vector dimension. In the formula, the inner product of each row vector of matrix **Q** and **K** is calculated. In order to prevent the inner product from being too large, it is divided by the square root of $d_{k}$. After **Q** is multiplied by the transpose of **K**, the resulting matrix can represent the attention intensity between vectors. After $QK^{T}$ is obtained, softmax is used to calculate the attention coefficient of each vector for other vectors. softmax in the formula is to softmax each row of the matrix, that is, the sum of each row becomes 1. After the softmax matrix is obtained, it can be multiplied by **V** to obtain the final output **Z**.

## 3 Method

### 3.1 Sequence-to-image

It is worth noting that the nanopore signal generated is a sequence of unequal length. Therefore, the unprocessed nanopore data cannot be directly applied as input for the deep learning model. To solve this issue, we propose the use of a S2I module that applies transformation rules to convert the unequal length sequence to a fixed-size image. These transformation rules involve the application of two main technologies, namely the GASF and GADF methods.

Suppose the time series is $X=\{x_{1},\ldots ,x_{n}\}$, the length of the series is *n*, we can use the normalization method to compress the time series to [0,1] or [-1,1]:

(4) $${\tilde{x}}_{0}^{i}=(x_{i}-\min(X))/(\max\left(X\right)-\min(X))$$

(5) $${\tilde{x}}_{-1}^{i}=(\left(x_{i}-\max(X)\right)+(x_{i}-\min(X)))/(\max\left(X\right)-\min(X))$$

where ${\tilde{x}}_{0}^{i}\in \left[0,1\right],\forall 1\leq i\leq n$,$\;{\tilde{x}}_{-1}^{i}\in \left[-1,1\right],\forall 1\leq i\leq n$. Trigonometric functions can then be used instead of normalized values. The following general ${\tilde{x}}_{i}$ represents the normalized time series, making $\phi_{i}=\mathrm{arc}\;\text{cos}\;\;{\tilde{x}}_{i}$,$\;{\tilde{x}}_{i}\in \left[-1,1\right],\;1\leq i\leq n$.Therefore, $\phi_{i}\in [0,\pi ]$, then $\sin\phi_{i}\geq 0$.

(6) $$\mathrm{GASF}={\left[\cos\left(\phi_{i}\right)\cdot \cos\left(\phi_{j}\right)-\sin\left(\phi_{i}\right)\cdot \sin\left(\phi_{j}\right)\right]}_{n\times n}$$

making $\tilde{X}={\left(\cos\left(\phi_{i}\right),\ldots ,\cos\left(\phi_{n}\right)\right)}^{T}$, the GASF can be changed as:

(7) $$\mathrm{GASF}=\tilde{X}\cdot {\tilde{X}}^{T}-\sqrt{I-{\tilde{X}}^{2}}\cdot \sqrt{I-{{\tilde{X}}^{T}}^{2}}$$

The above are all element multiplication and addition, and I represents the identity matrix. Its diagonal matrix is:

(8) $$\mathrm{diag}\left(\mathrm{GASF}\right)=\left\{\cos\left(2\phi_{1}\right),\ldots ,\cos\left(2\phi_{n}\right)\right\}=\left\{2\cos^{2}\left(\phi_{1}\right)-1,\ldots ,2\cos^{2}\left(\phi_{n}\right)-1\right\}={\left\{{\mathrm{GASF}}_{ii}\right\}}_{1\leq i\leq n}$$

If min-max normalization is used, the ${\tilde{x}}_{i}$ can be deduced from $\mathrm{diag}\left(\mathrm{GASF}\right)$, because $2{\tilde{x}}_{i}^{2}-1=2\cos^{2}\left(\phi_{i}\right)-1={\mathrm{GASF}}_{ii}$, then $y_{i}=\sqrt{({\mathrm{GASF}}_{ii}+1)/2}$.

Define the matrix GADF as:

(9) $$\mathrm{GADF}={\left[\sin(\phi_{i}-\phi_{j})\right]}_{1\leq i,j\leq n}={\left[\sin\left(\phi_{i}\right)\cdot \cos\left(\phi_{j}\right)-\cos\left(\phi_{i}\right)\cdot \sin\left(\phi_{j}\right)\right]}_{1\leq i,j\leq n}=\sqrt{1-X^{2}}\cdot X^{T}-X\cdot \sqrt{1-{\left(X^{T}\right)}^{2}}$$

Supposed *n* is the length of the input sequence, $X=\{x_{1},\ldots ,x_{n}\}$ is the input sequence. Then *l* is the value of the size of the output image (ie, the image is l×l). We use the GADF or GASF to obtain the transformed image set IMG_GADF_ or IMG_GASF_. The image dataset is used to train and test the model to achieve the goal of high-precision identification.

The operational mechanism of the S2I transformation primarily comprises four key steps: (i) Scaling the Input Sequence: The initial step involves scaling the input sequence to a standardized length, ensuring uniformity across various sequences. (ii) Normalizing the Scaled Sequence: Subsequently, the scaled sequence is normalized to fit within the range [0, 1], facilitating consistent data handling and analysis. (iii) Generating Polar Coordinates: The next step involves transforming the normalized sequence into polar coordinates. This transformation employs the time component of sequence as the radius, while utilizing the inverse cosine of the scaled sequence value as the angular element. (iv) Applying GADF or GASF Formula: The derived polar coordinates are then subjected to the GADF or GASF formula. This process computes and generates a two-dimensional matrix, serving as the subsequent model input. The datasets utilized in this article include the RNA classification dataset and the ONT barcode dataset. Both datasets exhibit distinct morphological differences in sequence patterns across different categories. After passing through the S2I transformation steps, especially during the conversion into polar coordinates, these morphological differences between different categories persist. This retention of fundamental sequence characteristics is instrumental in ensuring that even after transformation into images, the inherent one-dimensional characteristics of the sequences are extended into two dimensions. This extension to two-dimensional representations enables subsequent deep learning network models to achieve superior classification outcomes. The transformation into images enhances the ability of model ability to capture and interpret intricate sequence patterns, thereby facilitating more effective and accurate classification within the dataset.

The objective of the S2I module is to convert sequences of unequal lengths into images of uniform dimensions. The transformed images ought to exhibit clear distinctions between different categories while maintaining uniform color distribution within categories. The S2I module incorporates GASF and GADF as critical components. Machine learning systems are inherently optimal for processing image data. Ultimately, utilizing a deep learning network that conducts layer-by-layer computational iterations can further improve classification accuracy. The transformed images are a testament to the efficacy of our S2I approach, which enables the transformation while retaining clear distinctions between different sequence categories.

### 3.2 Example of S2I module

In Fig. 1a, a highly representative RNA nanopore sequence and ONT barcode nanopore sequence are depicted with different colors highlighting various categories. The transformation process of GASF or GADF is exemplified in Fig. 1b, entailing polar coordinate transformation. Fig. 1c shows RNA and ONT barcode datasets transformed by the S2I module. A comparison between Fig. 1a and Fig. 1c for the RNA dataset demonstrates apparent disparities between original sequences of different categories, with some having identical shapes but different sequenced current amplitudes. Fig. 1d demonstrates a concise illustration of the S2I transformation process. In this example, a sequence of length 10 undergoes several transformation steps. Initially, the sequence is aggregated and reduced in size using the Piecewise Aggregation Approximation method. Following this reduction, the sequence is scaled to fit within the interval [0, 1]. Subsequently, polar coordinates are generated by associating the time values of the sequence with the radius, while the inverse cosine of the scaled sequence values is employed as the angular component. These polar coordinates serve as the basis for the subsequent transformation steps. The next step involves the GADF process. Here, the angles derived from the polar coordinates are subjected to subtraction and combination operations. The resulting values are then processed using the cosine function to construct a matrix representation. This matrix serves as the output, representing the final image resulting from the S2I transformation process illustrated in Fig. 1d. The S2I conversion preserves the crucial basis for discriminating category differences in the original sequence and is conducive to subsequent deep learning models. In the ONT barcode dataset, significant peaks differentiate between categories and serve as the sole basis for classification, necessitating their preservation in the transformed image. The position of the peak in polar coordinates affects the color weighting of image, preserving vital information in the S2I transformed image for efficient recognition by subsequent deep learning models.

### 3.3 T-S2Inet

**Figure 2.**
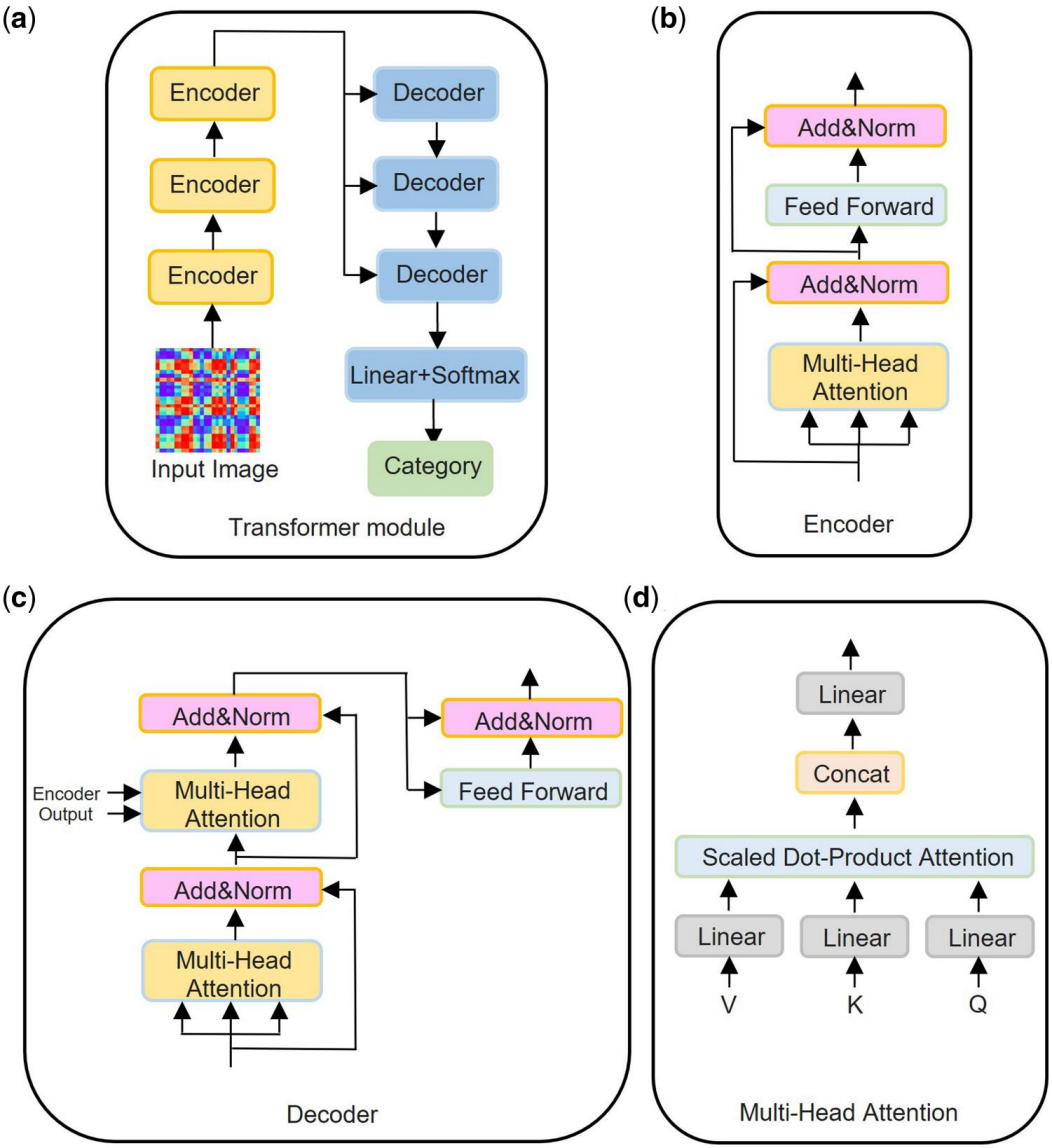
The schematic diagram of each important module of T-S2Inet. (a) The diagram of the Transformer module. (b) The diagram of the Encoder module. (c) The diagram of the Decoder module. (d) The diagram of the Multi-Head Attention module.

Utilizing the S2I module, we conduct T-S2Inet analysis for the prediction of RNA types and barcode types. During data preprocessing, the S2I module transforms the initial input sequence into an image (illustrated in Fig. 1c). In RNA datasets, the variation in sequences is primarily manifested in the form of the sequence output and the temporal relationship, as such, cannot be used as a discriminant basis for classification. It is challenging to determine the specific type of a sequence solely based on the temporal relationship. In this work, the transformed images do not possess temporal relationships, giving the Transformer module an advantage over other neural networks for local pattern recognition (LeCun and Bengio 1995, LeCun *et al.* 2015). The main structure of our model is an encoder and a decoder, with 3 layers of encoder and 3 layers of decoder. After the decoder, a linear fully connected layer plus an activation function output category is connected. The encoder module consists of three parts, the multi-head attention module is used for the S2I converted sequence, and “Add&Norm” and “Feed Forward” are applied to the output of the multi-head attention module. Hyperparameters used in the encoder layer: hidden layer size 32, number of heads 2, dropout set to 0.5. The Decoder module also consists of three parts, the two multi-head attention modules and the “Add&Norm” module are connected in series and connected to the decoder input. The output of the encoder is linked to the second multi-head attention module. Secondly, “Add&Norm” and “Feedforward” are connected to the output of the second “Add&Norm” module. Hyperparameters used in the decoder layer: hidden layer size 32, number of heads 2, dropout set to 0.5. The particular structure of Transformer module presented in Fig. 2a, consisting of an Encoder and a Decoder. The Encoder is shown in Fig. 2b. The Decoder module is shown in Fig. 2c. To capture the critical regions of the transformed image, we use the Transformer module, with the Multi-Head Attention module being the most significant segment, as shown in Fig. 2d. The inputs for the attention module are matrices Q, K, and V, and the Scaled Dot-Product Attention is utilized for the three inputs. Concat is applied to the Scaled Dot-Product Attention output, ensuring that the input and output have corresponding dimensions.

This article follows the classic Transformer network model. Both the encoder layer and decoder layer comprise three layers, and the input data is the image data obtained after S2I transformation. To minimize the exploitation function, we adopted the Adam optimization algorithm (Kingma and Ba 2014) (LR = 0.001; decay =0.97; batch size of 256). For RNA molecular classification datasets comprising 2775 samples, we performed cross-validation, with the training dataset randomly divided into a training subset and a validation subset for the model parameter training and validation. The training dataset for the ONT barcode dataset contains 52525 samples, with the validation dataset comprising 2189 samples and the testing dataset containing 3464 samples.

## 4 Experiments

In our experiments, we assess the generalization capability and efficacy of our T-S2Inet on the RNA nanopore dataset for predicting the type of RNA molecules. We compare it with previous baseline models (Guan *et al.* 2022). To evaluate the efficiency of the S2I module and verify its universality on other nanopore data, we conduct an additional experiment using the ONT barcode data from the study (Misiunas *et al.* 2018).

### 4.1 Dataset

This study utilizes two distinct nanopore datasets, each presenting unique characteristics essential for the proposed research. The first dataset, termed the RNA dataset, is primarily gathered via the MspA nanopore technology. This dataset encapsulates six RNA analytes: overhanged siRNA, two variations of blunt siRNA, two forms of tRNA, and 5s rRNA, complemented by a single noise signal category. Notably, these diverse RNA analytes exhibit substantial variations in their sequence lengths, leading to the accumulation of extensive, continuous sequences. To effectively handle these prolonged sequences and ensure comprehensive data utilization, a segmentation algorithm was indispensably employed. This algorithm was instrumental in truncating the raw RNA sequences, thereby obtaining a complete dataset conducive to analysis. The RNA dataset, consisting of 2775 samples as detailed in reference (Guan *et al.* 2022), serves a crucial role in verifying the substantial performance enhancements attributed to the proposed methodology.

In parallel, the second dataset, designated as the ONT barcode dataset, comprises a training subset with 52 525 samples, a testing subset containing 3464 samples, and a validation subset comprising 2189 samples. This dataset embodies eight distinct code categories ranging from “000” to “111”. The rationale behind selecting these two datasets lies in their inherent characteristics, their unequal sequence lengths. The convolutional neural network cannot be directly applied due to these variations. To address this challenge, this paper introduces a S2I network, specially designed to convert these disparate sequences into equal-sized images. This transformation enables the subsequent application of a convolutional neural network, facilitating effective learning and analysis.

To maintain input length consistency crucial for neural network operations, our methodology integrates the proposed S2I module. This innovative module serves as a pivotal component, facilitating the transformation of 1D sequences into structured 2D images. Specifically, both the RNA dataset and the ONT barcode dataset undergo this transformation process. Resultantly, these disparate 1D sequences are converted into standardized 64x64 pixel images. This transformation into 2D images holds paramount significance, particularly in optimizing the performance of subsequent analyses within the experimental framework. Through rigorous testing and evaluation, it has been empirically established that employing this S2I module to generate 64 × 64 images from the RNA dataset and the ONT barcode dataset yields the most promising and optimal results in our experimental analyses.

Experimental settings and measurement indicators can be seen in Supplementary Section A.

### 4.2 Experimental results on RNA dataset

This paper adopts several baseline methods drawn from previous studies (Wang *et al.* 2021) and traditional machine learning algorithms. These methods encompass Classification and Regression Trees (CART) (Lawrence and Wright 2001), Xgboost (Chen and Guestrin 2016), Random Forest, KNN (Guo *et al.* 2003), and Gradient Boost (Ke *et al.* 2017). The selection of these comparison methods is rooted in our prior work, specifically the utilization of these baseline methods in our previous paper, S2Snet (Guan *et al.* 2022). The rationale behind choosing these methods lies in their applicability to the characteristics of the RNA dataset, which exhibits unequal sequence lengths, rendering deep learning methods impractical without sequence conversion. Consequently, traditional machine learning techniques are preferred for their compatibility with such data attributes.

Among the selected baselines, S2Snet (Guan *et al.* 2022) is a sequence-to-sequence network that leverages deep learning methods. Its inclusion in the baseline methods facilitates a more meaningful comparison with the methodology proposed in this paper, thereby allowing for a comprehensive assessment of the effectiveness of proposed method.

Initially, we validate the ultimate performance of the proposed T-S2Inet technique. The findings exhibit that T-S2Inet accurately identifies nearly all events, including highly intricate shapes, as illustrated in Fig. 1a. Table 1 presents a quantitative comparison of accuracy.

**Table 1.** Performance Comparison between T-S2Inet and other baseline algorithms.

Method	Accuracy	Recall	F1 Score	Speed/average
KNN	0.878	0.852	0.850	1m2s
CART	0.895	0.868	0.867	1m18s
GradientBoost	0.909	0.892	0.893	3m5s
Xgboost	0.928	0.917	0.915	8m57s
Random Forest	0.934	0.932	0.934	10m48s
S2Snet^a^	0.951	0.947	0.947	35m43s
**T-S2Inet** ^a^	**0.973**	**0.971**	**0.971**	47m21s

a Indicates that the two methods do the *t*-test for significant difference analysis, the *P*-value is 5.86 × 10^−14^<0.01. The Bold indicates the performance is better than other method.

T-S2Inet attains a remarkable accuracy of 0.973 for RNA type classification; this performance surpasses the accuracy of all previous algorithms, including S2Snet, which achieved an accuracy of 0.951 in this dataset configuration. Moreover, the recall and F1 Score are identical to the accuracy. The last column of Table 1 depicts the average total training and testing time. The selection of accuracy, F1 Score, and recall serves as effective metrics for assessing the proficiency of model in classifying sequences of unequal lengths. Recall, specifically, is instrumental in gauging the number of missed samples that were not predicted by the model. On the other hand, the F1 Score provides an evaluation of the capability of model to accurately identify positive examples within the dataset. In addition to these performance metrics, the time taken for model training is a crucial factor used to determine the computational cost associated with training the model. This time-based assessment is vital in comprehensively evaluating the efficiency of model, particularly in relation to the computational resources required for its training process. Although the time cost of T-S2Inet is slightly higher than S2Snet, our model is based on the Transformer mechanism, which requires more training time to capture local patterns.

**Figure 3.**
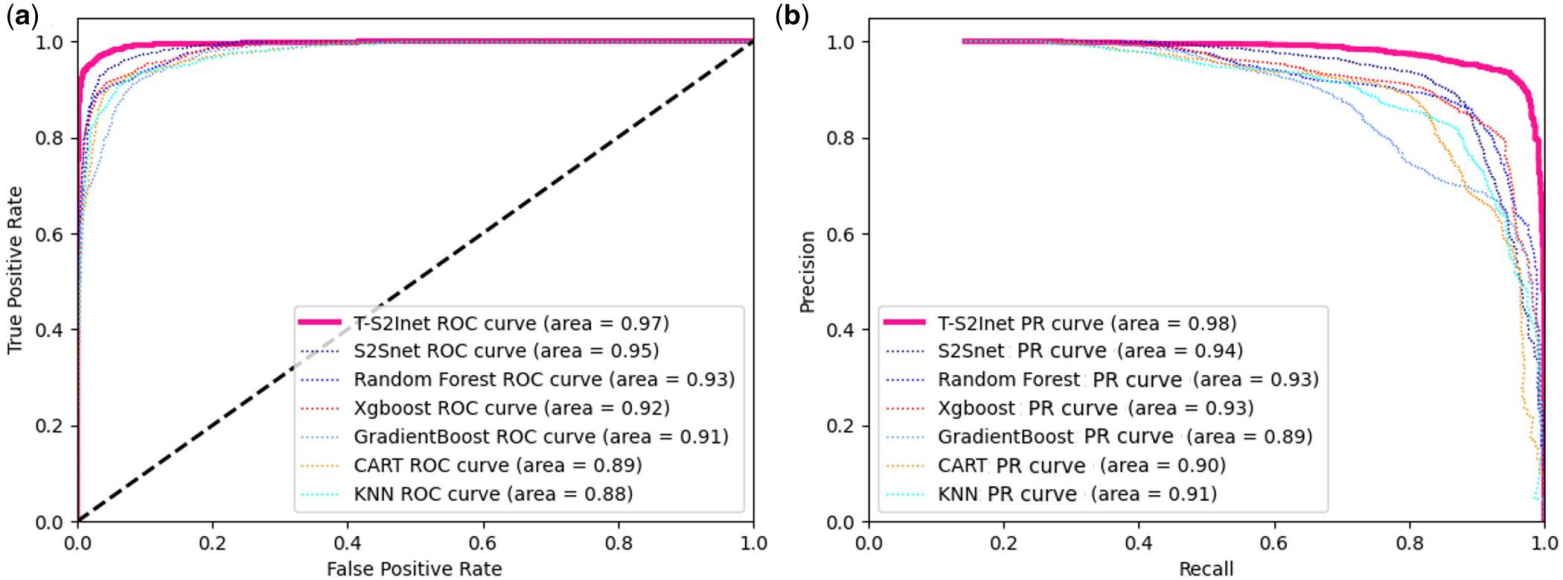
The ROC-PR curve on RNA dataset. (a) This is the ROC curve result. (b) This is the PR curve result. The deep pink color represents the results of T-S2Inet proposed in this article.

Figure 3 shows the ROC-PR curve results of several baseline methods. Since the dataset is a multi-classification problem, for the convenience of observation, only the micro average results in “sklearn” are used to represent the classification performance. It can be seen from the figure that our proposed method T-S2Inet has obvious advantages over other baseline methods in terms of ROC and PR curves.

**Figure 4.**
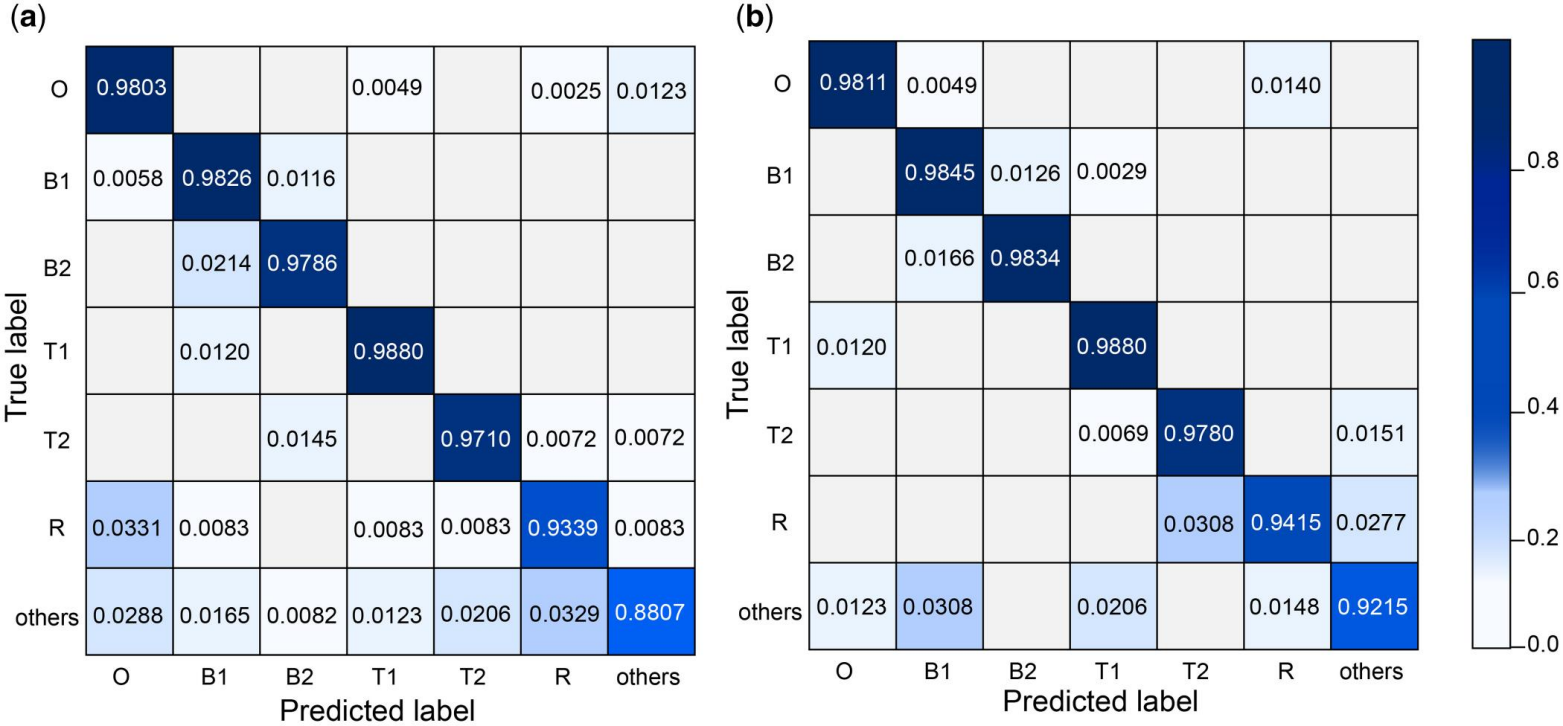
The diagram of the experimental result on RNA dataset. (a) The confusion matrix results of S2Snet classification on the RNA dataset. (b) The confusion matrix results of the T-S2Inet classification on the RNA dataset. The RNA types are overhanged siRNA (O), blunt siRNA type 1 (B1), blunt siRNA type 2 (B2), tRNA type 1 (T1), tRNA type 2 (T2), 5S rRNA (R).

Figure 4a and b show the confusion matrix results of S2Snet and T-S2Inet. The results of the previous S2Snet method are shown in Fig. 4a. The accuracy of the overhanged siRNA, blunt siRNA type 1 and siRNA type 2, tRNA type 1 and type 2, and 5S rRNA are 0.9803, 0.9826, 0.9786, 0.9880, 0.9710, and 0.9339, respectively. Significantly, the result of the T-S2Inet method in Fig. 4b that the accuracy of overhanged siRNA, blunt siRNA type 1 and siRNA type 2, tRNA type 1 and type 2, and 5S rRNA are 0.9811, 0.9845, 0.9834, 0.9880, 0.9780, and 0.9415, respectively.

The performance of T-S2Inet is better than that of the previous S2Snet method. In addition, the classification performance of overhanged siRNA, tRNA type 2, blunt siRNA type 1, blunt siRNA type 2, tRNA type 1, and 5S rRNA is significantly improved because the transformed sequences are clearly different from each other. In particular, these RNA raw sequences have the same shape, which is rather indistinguishable using the classical machine learning method. The Transformer module can capture critical feature information. The critical features reflect the property of the RNA molecule. The classification performance of “others” is slightly increased.

**Figure 5.**
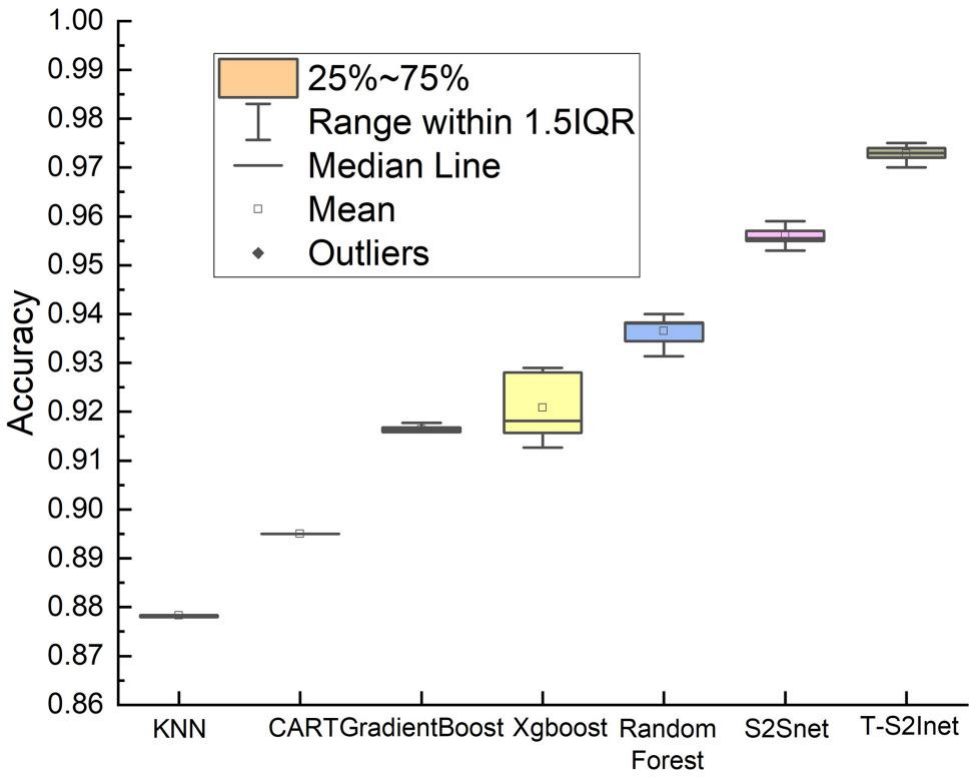
The experimental result of the box plot. Each one contains ten results, and the horizontal axis is KNN, CART, GradientBoost, Xgboost, Random Forest, S2Snet, and our method T-S2Inet, respectively.

In addition, it is interesting to analyze the distribution of the results (i.e. accuracy) of the different methods, as shown in Fig. 5. The horizontal axis shows KNN, CART, GradientBoost, Xgboost, Random Forest, S2Snet and T-S2Inet, respectively. For RNA type classification, we run all methods 10 times and calculate the statistical results such as mean and standard. For all baseline methods, the accuracy is lower than T-S2Inet. Our method has relatively low variation, which guarantees its robustness. This box plot again shows that T-S2Inet is an effective method to achieve the best performance in distinguishing RNA molecule type from mixed analyte.

**Figure 6.**
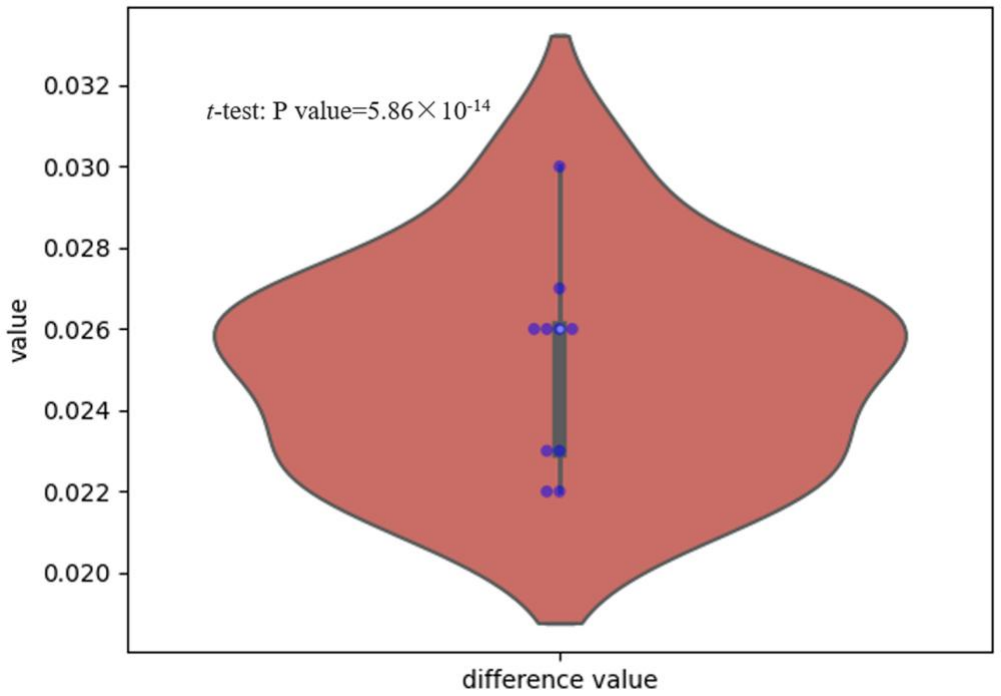
The experimental result of T-S2Inet improves accuracy relative to S2Snet on the RNA dataset. The result verifies a significant difference by the *t*-test experiment..

We perform the *t*-test (Boneau 1960) between the results of S2Snet and T-S2Inet. First, we perform ten-fold cross-validation in the experimental phase and obtain two vectors of length ten, which we call *V*_S2S_ and *V*_S2I_, respectively. The differences between the two vectors are shown in Fig. 6. Second, we perform the Kolmogorov–Smirnov test (Massey Jr 1951) to determine whether the two vectors follow the normal distribution. For the hypothesis, we use the setting most commonly used in statistical analysis. We accept the hypothesis if the normal distribution is satisfied with *P* > .05, otherwise reject the hypothesis with *P* < .05. The Kolmogorov–Smirnov test for vectors *V*_S2S_ and *V*_S2I_ is 0.83 and 0.45, respectively, indicating that the two vectors follow the normal distribution. Finally, we perform the *t*-test to determine whether *V*_S2S_ and *V*_S2I_ have a significant difference. In the *t*-test, we accept H0 (no significant difference) if *P* > .05, otherwise we accept H1(significant difference) with *P* < .05. In our experimental results, the *P*-value of the *t*-test between *V*_S2S_ and *V*_S2I_ is 5.86 × 10^−14^, indicating that they are significantly difference. Moreover, the mean value of our result (*V*_S2I_) is larger than that of S2Snet (*V*_S2I_), indicating that our proposed method can achieve higher classification accuracy.

### 4.3 Experimental results on ONT barcode dataset

**Figure 7.**
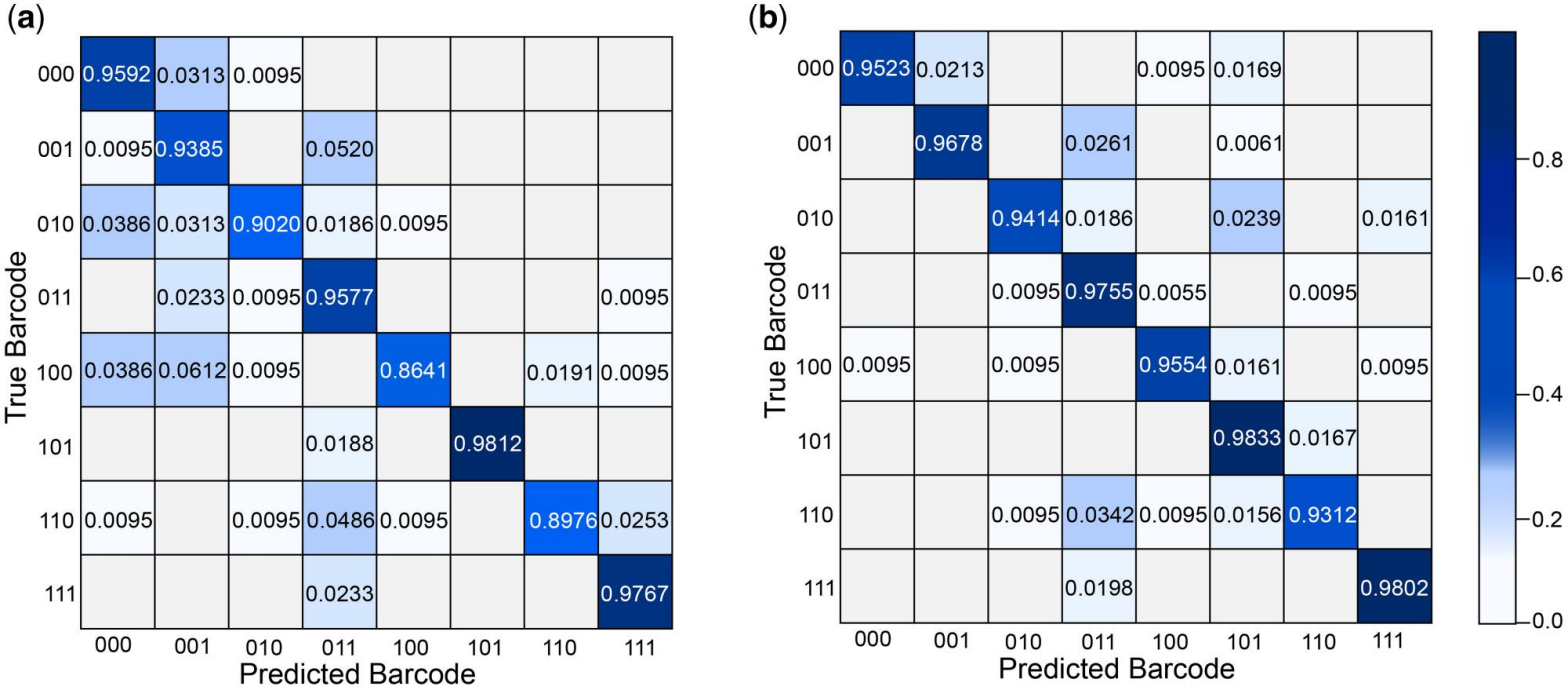
The experimental result on ONT barcode dataset. (a) The confusion matrix results of QuipuNet classification on the ONT barcode dataset. (b) The confusion matrix results of the T-S2Inet classification on the ONT barcode dataset.

The ONT barcode dataset was extracted from the paper (Misiunas *et al.* 2018). The reported accuracy was 0.946, and the corresponding confusion matrix is presented in Fig. 7a. A 700-element vector served as input, which was padded with Gaussian noise (*μ*  =  0, *σ*  =  0.072, representing the average noise level) at the end in cases of shorter sequences. To evaluate the effectiveness of our proposed T-S2Inet module for the ONT barcode dataset, we utilized the S2I module to transform the sequence into an image and applied the T-S2Inet on the transformed data. The baseline models for comparison included QuipuNet and S2Snet with S2S module as used in the article (Misiunas *et al.* 2018). The results of the experiment are detailed in Table 2.

**Table 2.** Performance Comparison between T-S2Inet and other baseline algorithms on ONT barcode dataset.

Method	Accuracy	Recall	F1 Score
QuipuNet	0.946	0.946	0.945
S2Snet	0.948	0.947	0.947
**T-S2Inet**	**0.962**	**0.961**	**0.960**

The Bold indicates the performance is better than other method.

The experiment demonstrated that our T-S2Inet model achieved a performance of 0.962, and the corresponding confusion matrix is presented in Fig. 7b. The performance was considerably improved compared to QuipuNet and S2Snet. Moreover, the experimental results align with the anticipated outcomes, confirming that our proposed T-S2Inet can be effectively applied in all nanopore devices. Regarding the “000” barcode, there was a slight decrease in classification performance. Compared with the QuipuNet method, some sequences of “000” are misidentified as “100” and “101”, resulting in a lower accuracy in identifying “000” than QuipuNet. The reason for this situation may be mainly because the sequence of “000” is similar to some images of “100” and “101” after being converted into an image, thus causing misclassification. Whereas the performance for barcodes “001”, “010”, “011”, “100”, “101”, “110”, and “111” was slightly improved. This is attributed to the ability of our T-S2Inet model, incorporating the Transformer module, to more accurately capture the essential features of these barcode sequences.

### 4.4 Experimental results on other two datasets

To verify the scalability, we use the other two nanopore datasets to implement the baseline strategy. The first dataset is from (Smith *et al.* 2015), the author proposed a novel strategy for barcoding and demultiplexing direct RNA sequencing nanopore data that does not rely on basecalling or additional library preparation steps. The dataset contains 160K samples for training, 40K samples for testing, and 40K samples for validation. Another dataset we use to check scalability from the paper (Wang *et al.* 2019). O^6^-carboxymethylguanine (O^6^-CMG) is a highly mutagenic alkylation product of DNA that induces transition mutations relevant to gastrointestinal cancer. First, we cut the original data of the nanopores used in the paper (Wang *et al.* 2019), and obtained a dataset of 1010 samples in total. To evaluate the scalability of T-S2Inet model on more nanopore datasets, we test the use of model with two datasets for different tasks. The experimental results are shown in Table 3. From the table, it can be seen that for the DeePlexiCon dataset, T-S2Inet can achieve the 96% classification performance, while for the O^6^-CMG dataset, T-S2Inet can achieve the 99% classification performance. The two datasets once again verify its significant performance improvement compared to previous methods. This experimental result also guarantees that the T-S2Inet is universal and has good scalability for the universal nanopore dataset.

**Table 3.** Performance Comparison between T-S2Inet and other baseline algorithms on DeePlexiCon and O^6^-CMG.

Method	DeePlexiCon	O^6^-CMG
	Accuracy*↑*	Accuracy*↑*
KNN	0.878 ± 0.002	0.965 ± 0.006
CART	0.883 ± 0.013	0.970 ± 0.014
GradientBoost	0.891 ± 0.041	0.973 ± 0.032
Xgboost	0.905 ± 0.034	0.975 ± 0.025
Random Forest	0.918 ± 0.012	0.980 ± 0.054
S2Snet^a^	0.953 ± 0.006	0.985 ± 0.029
**T-S2Inet** ^a^	**0.962 ± 0.011**	**0.991 **±** 0.014**

a Indicates that the two methods do the *t*-test for significant difference analysis, the *P*-value is 3.14 × 10^−6^<0.01. The Bold indicates the performance is better than other method.

### 4.5 Ablation study

**Figure 8.**
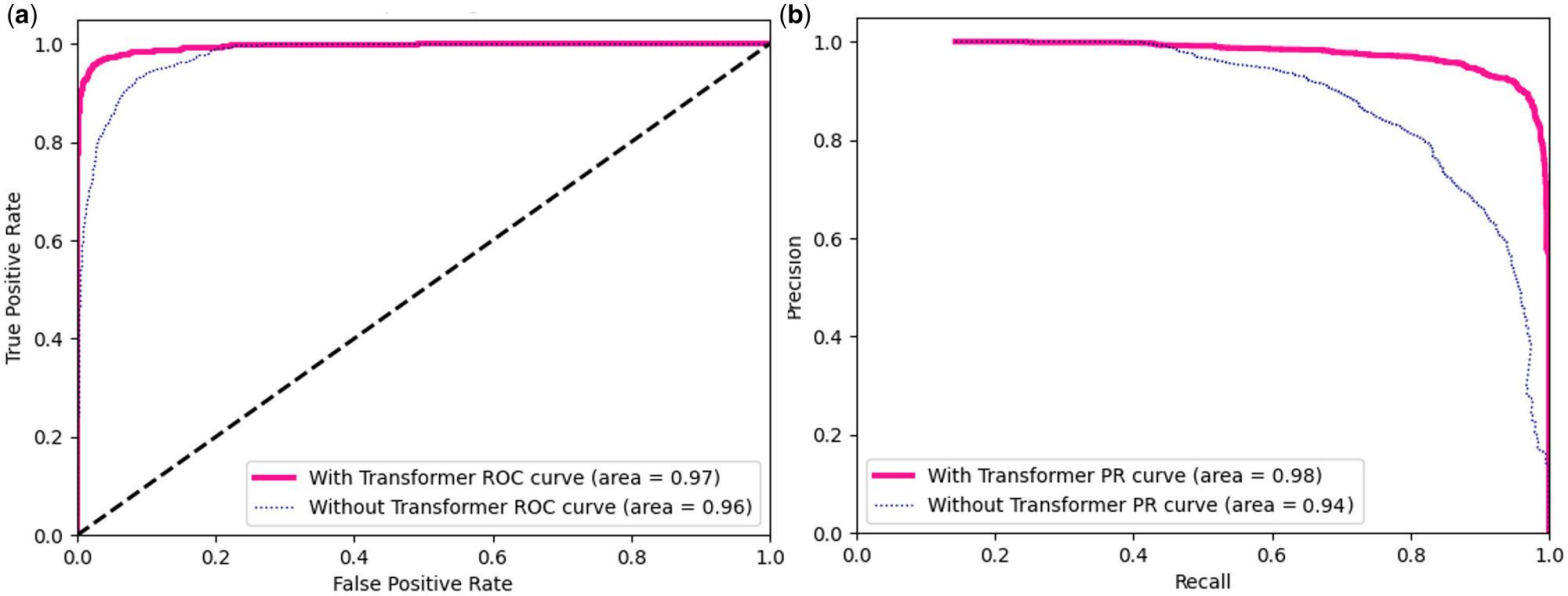
The ROC-PR curve of ablation study. (a) This is the ROC curve result. (b) This is the PR curve result.

T-S2Inet is a Transformer-based approach that comprises a crucial component—the Transformer module, which captures the local patterns of input images. We conducted an ablation study on the T-S2Inet model variants to demonstrate the efficacy of the module in enhancing the performance of nanopore sequence classification. The ablation design involved eliminating the encoder and decoder segments of the Transformer and employing four layers of standard convolution, followed by Linear and Softmax to achieve classification. The objective was to establish the superiority of the Transformer mechanism over convolution. The results are presented in Table 4, and the ROC-PR curve is shown in Fig. 8. Consequently, we draw the following conclusions: (i) The Transformer mechanism plays a pivotal role in enhancing the RNA type classification performance; (ii) The Transformer module is an indispensable component of our proposed T-S2Inet model and has a critical role in its efficacy.

**Table 4.** Ablation study results.

	Accuracy	Recall	F1 score
–	0.964	0.963	0.963
√	**0. 973**	**0.971**	**0.971**

The “√” express as the Transformer module is used, in contrast “-” express as the Transformer module is not used. The Bold indicates the performance is better than other method.

### 4.6 Selection of transformation strategy

T-S2Inet utilizes GASF/GADF for nanopore sequence transformation, and trains and predicts the model through a subsequent deep neural network. The transformation strategy can be used as GASF, GADF, MTF and RP. To compare the impact of different transformation strategy on test performance, we conducted experiments using four strategies in this section. The experimental results, presented in Table 5, indicate that the performance of data training following RP, MTF and GASF transformation is marginally lower than that of data training using GADF transformation. Consequently, in this article, we consistently employ GADF as a vital constituent of the T-S2Inet sequence transformation image model, unless otherwise specified.

**Table 5.** Comparison of results after four transformation strategies.

Strategy	Accuracy	Recall	F1 Score
T-S2Inet-RP	0.961	0.960	0.960
T-S2Inet-MTF	0.965	0.964	0.963
T-S2Inet-GASF	0.971	0.970	0.970
**T-S2Inet-GADF**	**0.973**	**0.971**	**0.971**

The “T-S2Inet-RP” means the data transformed by RP is used for T-S2Inet model training, the “T-S2Inet-MTF” means the data transformed by MTF is used for T-S2Inet model training, the “T-S2Inet-GASF” means the data transformed by GASF is used for T-S2Inet model training, “T-S2Inet-GADF” means the data transformed by GADF is used for T-S2Inet model training. The Bold indicates the performance is better than other method.

## 5 Discussion

This article introduces a novel deep learning network model, termed T-S2Inet, designed for the recognition of original nanopore sequences using a Transformer-based architecture. The initial step of this model involves converting unequal-length one-dimensional raw current signals. Among various analyzed methods, the GADF approach demonstrates superior performance in this conversion process. Subsequently, the equal-sized images generated are employed for learning and prediction within the deep learning network. In the validation process using the RNA dataset and ONT barcode dataset, our proposed T-S2Inet exhibits superior performance compared to prior models. The distinctive feature of our model lies in its utilization of the Transformer structure, enabling enhanced capture of crucial features within image blocks pivotal for classification performance. This design choice significantly enhances the learning and predictive capabilities of model. However, It is worth noting that the use of the Transformer structure leads to longer training times compared to traditional methods. Our model primarily addresses the challenge of unequal lengths within raw nanopore current signals. We propose a solution that yields promising results in handling this issue. One aspect that requires further improvement in this paper pertains to incorporating interpretability analysis. Future work will concentrate on enhancing interpretability analysis within the model.

## 6 Conclusion

This study presents a novel approach based on the Transformer module, termed T-S2Inet, for classifying the original nanopore detection signal obtained from nanopore devices. Our findings demonstrate that T-S2Inet outperforms prior studies in terms of classification performance. The proposed method effectively addresses the issue of unequal sequence lengths in deep learning of MspA and Oxford nanopore devices, but is intended solely for analyses of original nanopore detection signals. We validate the efficacy of our strategy using RNA molecular classification and ONT barcode datasets. Specifically, this article investigates the utility of our proposed sequence transformation approach and evaluates its impact on traditional Transformer networks. In follow-up work, we aim to develop a dedicated deep learning model for transformed data that enhances the accuracy of nanopore detection signal recognition. It is worth noting that the concept of sequence transformation shows promise for advancing bioinformatics research in the field of nanopore technology.

## Supplementary Material

btae083_Supplementary_Data

## Data Availability

The data underlying this article will be shared on reasonable request to the corresponding author.
