# Supplementary material for: T-S2Inet: Transformer-based sequence-to-image network for accurate nanopore sequence recognition
Source: Bioinformatics. 2024 Feb 15;40(2):btae083. doi: 10.1093/bioinformatics/btae083 (PMC10902682; doi:10.1093/bioinformatics/btae083)
Supplement: btae083_Supplementary_Data [file btae083_supplementary_data.zip › TableS1.docx]

**Table S1. Performance Comparison between different parameter of the dropout module.**

| Parameter | 0.1 | 0.2 | 0.3 | 0.4 | 0.5 |
| --- | --- | --- | --- | --- | --- |
| Accuracy | 0.970 | 0.971 | 0.973 | 0.972 | 0.970 |
