# Supplementary material for: T-S2Inet: Transformer-based sequence-to-image network for accurate nanopore sequence recognition
Source: Bioinformatics. 2024 Feb 15;40(2):btae083. doi: 10.1093/bioinformatics/btae083 (PMC10902682; doi:10.1093/bioinformatics/btae083)
Supplement: btae083_Supplementary_Data [file btae083_supplementary_data.zip › T-S2Inet-SI.docx]

**Supporting Information**

# Experimental Settings

The size of S2I transform image is set as 64x64, which is suitable for the input of the Multi-Head Attention module of Transformer network. The parameter of the dropout module is set as 0.3, which is the probability of the inactive state. We chose ten commonly used dropout parameters, and the experimental result shows in Table 1 and 0.3 has the best performance.

The model is trained for 2000 epochs on a GPU (Nvidia GeForce GTX 2080 TI). To minimize the loss function, we use the Adam optimization algorithm [41] (LR = 0.001; decay =0.97; batch size of 256).

**Table S1. Performance Comparison between different parameter of the dropout module.**

| Parameter | 0.1 | 0.2 | 0.3 | 0.4 | 0.5 |
| --- | --- | --- | --- | --- | --- |
| Accuracy | 0.970 | 0.971 | 0.973 | 0.972 | 0.970 |

To evaluate the classification performance of the T-S2Inet, we use the classification accuracy, which is calculated by the quotient of correctly classified samples and total samples. For the binary classification, the samples can be divided into two categories according to their real categories and classifier prediction categories: true category is positive, predicted category is positive (True Positive, *TP*), true category is negative, predicted category is positive (False Positive, *FP*), true category is positive, predicted category is negative (False Negative, *FN*) and true category is negative, predicted category is negative (True Negative, *TN*).

The Accuracy is defined as:

$Acc=\frac{TP+TN}{total}$ (1)

Where *total* is the total number of samples. There is another metric to assess the classifier performance is the *precision*. It is defined as the quotient of *TP* and *TP+FP*, the computational formula is:

$P=\frac{TP}{TP+FP}$ (2)

The recall *R* is the quotient of *TP* and *TP+FN*, the computational formula is:

$R=\frac{TP}{TP+FN}$ (3)

Since, the F1 score is defined as:

$F1=\frac{2\times P\times R}{P+R}$ (4)

Specially, in this manuscript, the classification task is multi-class problem, the number of categories is seven which contains six kinds of RNA molecule type sequence and one noise sequence. For the multi-class problem, we use the macro-averaging that calculating the precision, recall and F1 of each type respectively, and then calculate the average value.

# Size of Transform Image

The dimensions of the transformed image exert a decisive influence on the overall performance of the final classification. Prior to deep learning model training, it is imperative to transform the original sequence, com-prising unequal lengths, into an image of equal proportions that adheres to deep learning requirements. In this experimental study, we established five different image dimensions for transformation, specifically 32, 64, 128, 256, and 512. The image size for transformation is attuned to the Transformer input size. The accuracy of varying image sizes is demon-strated in Fig. S1.

It is observed from the figure that the test accuracy is optimal when the image dimensions for transformation are 64. In the case of dimen-sions 128, 256, and 512, the test accuracy drops significantly, owing to the large size that results in loss of vital classification information. Con-versely, when the image dimensions are 32, the test accuracy is relatively low, as the small size inadequately captures crucial information. Conse-quently, the dimensions for transformation in this study are set at 64x64.


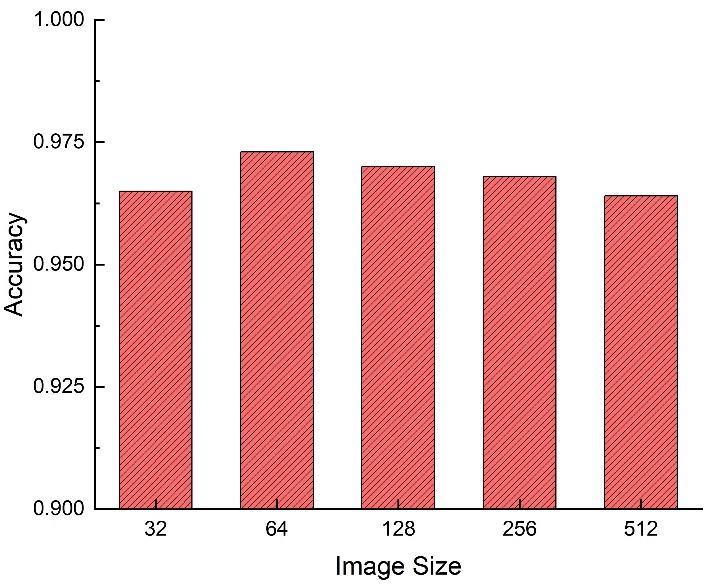
**Fig. S1.** **Accuracy of the different transformed image size.**
