# Supplementary figures and images for: T-S2Inet: Transformer-based sequence-to-image network for accurate nanopore sequence recognition
Source: Bioinformatics. 2024 Feb 15;40(2):btae083. doi: 10.1093/bioinformatics/btae083 (PMC10902682; doi:10.1093/bioinformatics/btae083)

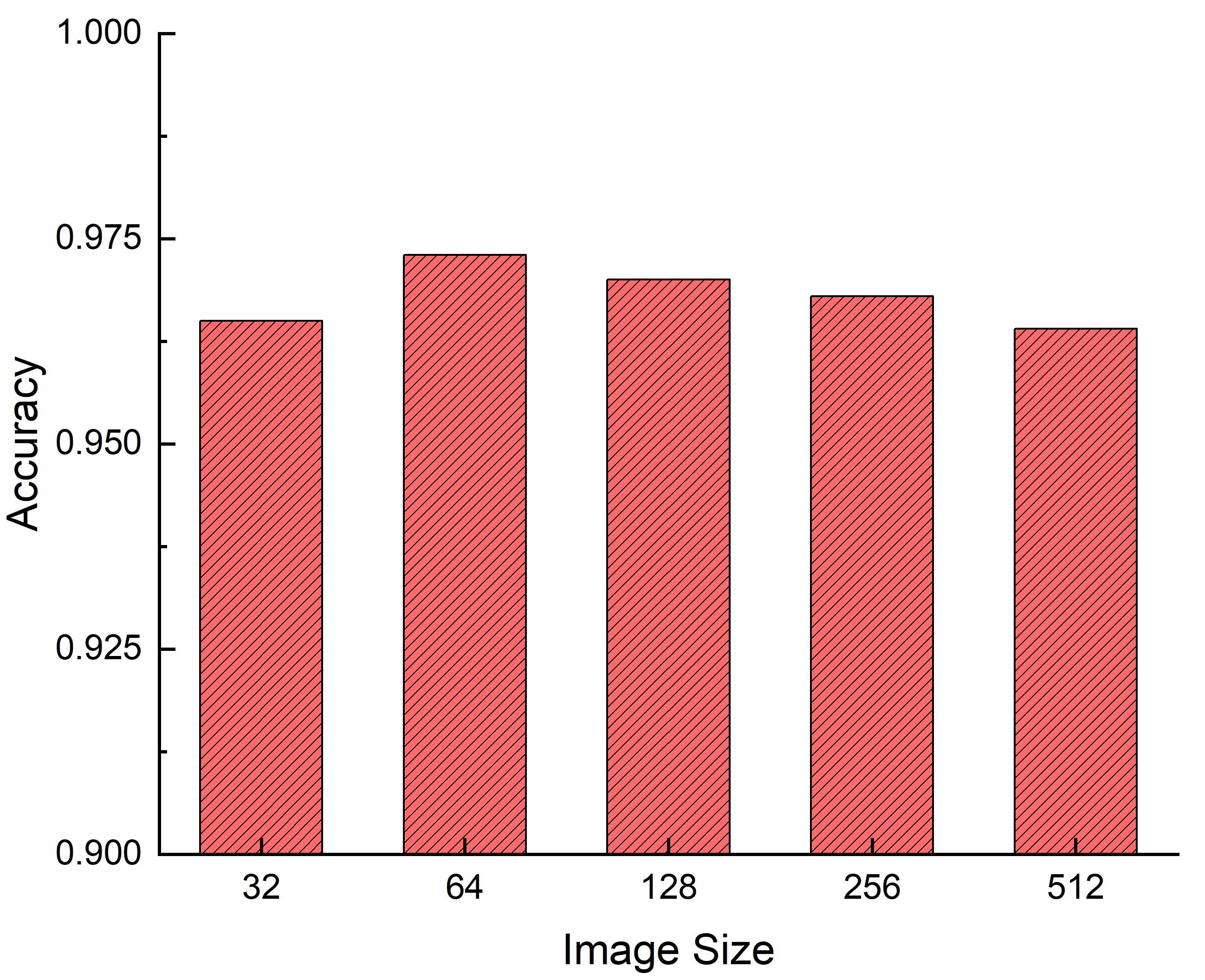

Supplement: btae083_Supplementary_Data [file btae083_supplementary_data.zip › figS1.jpg]
